# Strengthening Capacity for Prostate Cancer Early Diagnosis in West Africa Amidst the COVID-19 Pandemic: A Realist Approach to Rethinking and Operationalizing the World Health Organization 2017 Guide to Cancer Early Diagnosis

**DOI:** 10.5334/aogh.3519

**Published:** 2022-05-10

**Authors:** Elochukwu Fortune Ezenwankwo, Daniel A. Nnate, Catherine Adebukola Oladoyinbo, Hassan Mohammed Dogo, Ademola Amos Idowu, Chimdimma Peace Onyeso, Chidiebere Ndukwe Ogo, Motolani Ogunsanya, Olufikayo Bamidele, Chukwudi A. Nnaji

**Affiliations:** 1 Division of Physiological Sciences, Department of Human Biology, Faculty of Health Sciences, University of Cape Town, Anzio Road, Observatory, Cape Town 7925, South Africa; 2 African Behavioral Research (ABeR) Center, Federal University of Agriculture, Abeokuta, Nigeria; 3 Cancer Research Initiative, Faculty of Health Sciences, University of Cape Town, Anzio Road, Observatory, Cape Town 7925, South Africa; 4 Department of Nursing and Community Health, School of Health and Life Sciences, Glasgow Caledonian University, UK; 5 Department of Nutrition and Dietetics, Federal University of Agriculture, Abeokuta, Nigeria; 6 Department of Surgery, Urology Division, University of Maiduguri, Maiduguri, Nigeria; 7 Department of Chemical Pathology, Ekiti State University College of Medicine, Ado-Ekiti, Nigeria; 8 Military Hospital, GRA, Port Harcourt, Rivers State, Nigeria; 9 Department of Surgery, Federal Medical Centre Abeokuta, NG; 10 African Behavioral Research (ABeR) Center, Federal Univeristy of Agriculture, Abeokuta, Nigeria; 11 College of Pharmacy, University of Oklahoma Health Sciences Center, 1110 N. Stonewall Ave, Oklahoma City, US; 12 Institute for Clinical and Applied Health Research, Hull York Medical School, R341, University of Hull, UK; 13 Cochrane South Africa, South African Medical Research Council, Francie Van Zijl Drive, Parow Valley, Cape Town 7501, South Africa

## Abstract

Two years after SARS-CoV-2 (COVID-19) was declared a global public health emergency, the restoration, at least, to the pre-pandemic level of early diagnostic services for prostate cancer has remained enormously challenging for many health systems, worldwide. This is particularly true of West Africa as the region grapples also with the broader impacts of changing demographics and overly stretched healthcare systems. With the lingering COVID-19 crisis, it is likely that the current trend of late prostate cancer diagnosis in the region will worsen with a concomitant increase in the burden of the disease. There is, therefore, a compelling need for innovative and evidence-based solutions to de-escalate the current situation and forestall the collapse of existing structures supporting early prostate cancer diagnosis in the region. In this viewpoint, we make a case for the operationalization of the World Health Organization (WHO) guide to early cancer diagnosis to strengthen the capacity for early prostate cancer diagnosis in West Africa using a realist approach, drawing on participatory health research and evidence-based co-creation. Ultimately, we demonstrate the potential for developing COVID-19 responsive and context-specific models to optimize patient navigation/journey along the essential steps of the World Health Organization guide to early cancer diagnosis.

## Introduction

In response to the growing threats of the COVID-19 pandemic, healthcare authorities worldwide advocated for the suspension or downscaling of relatively low-to-moderate risk urologic oncology services [1,2,3]. Consequently, the world saw a tremendous reduction in prostate cancer-related early diagnostic services, including prostate-specific antigen tests, digital rectal examinations, prostate biopsies, radiological investigations, diagnostic staging, etc., and a concomitant decrease in the rate of prostate cancer diagnosis [4,5,6]. For instance, data from the Swedish National Prostate Cancer Register showed a 36% drop in the number of men diagnosed with prostate cancer in the first wave of the COVID-19 crisis in Sweden [7]. Likewise, clinical activities related to prostate cancer in Italy, including prostate biopsies, were reported to have reduced significantly between March 2020 and March 2021 compared with the previous year [8]. Across Europe, prostate biopsies were reduced by 62%, reaching a mean reduction of 12 procedures per month [9]. In West Africa, the extent to which the COVID-19 pandemic has impacted prostate cancer early diagnosis remains broadly speculative—largely due to lack of adequate data infrastructure and poorly developed integrated care pathway and surveillance system [10]. However, with the lingering COVID-19 crisis or as an aftermath, it is expected that the current trend in late prostate cancer diagnosis will worsen remarkably, with a consequent increase in late-stage presentation, emergency hospital admission, poor treatment outcome, lower relative survival and higher mortality rates.

There is a compelling need to explore evidence-based solutions to de-escalate the current situation and forestall the collapse of existing service systems supporting prostate cancer diagnosis in the region [11,12,13]. Although, currently, very limited data exist on the epidemiology and burden of prostate cancer in West Africa due to the limited availability of epidemiological evidence sources and population-based cancer registries, global cancer statistics and several genomic studies have pointed to prostate cancer as a rapidly growing public health epidemic in West Africa with far-reaching consequences for men in the region [14,15,16,17,18,19]. Current reported prevalence, for instance, ranges from 1 046 to 70 000 cases per 100 000 population, with estimated 55.7–64.6% 5-year relative survival [10]. Even with a slight decrease in the age-standardized incidence-to-death ratio between 2018 and 2020, the region experienced an increase (by 1.6) in the number of deaths per 100 000 in the short period [14,20].

Currently, several hospital-based studies are reporting between 33% and 50% of metastatic disease at diagnosis at a time when evidence is showing that nations with higher levels of emergency diagnoses have lower survival for most cancers [10,13,10,21,22,23]. Coupled with a growing incidence of late-stage prostate cancer diagnosis, the region, like the rest of sub-Saharan Africa is increasingly affected by more aggressive forms of the disease, with a higher likelihood of experiencing faster disease progression and worse survival outcomes [10,14,15,24,25]. With a cumulative risk-adjusted mortality of 2.13, deaths from prostate cancer in West Africa are among the highest in the world [14]. Even as the roles of biological, sociodemographic, environmental, and behavioral factors underlying this trend have been speculated [10,26], the current situation is complicated largely by health systems inadequacies typified by limited access to early diagnosis and substandard treatments [14,15,25]. Access to definitive diagnostic modalities such as biopsy or tumor staging is highly limited, as is access to both definitive and non-curative treatment options [10,13]. Other challenges include limited funding, scarcity of highly trained workforce, lack of region-specific guidelines, and lack of integrative population-based cancer care registry, among other barriers [10,13].

To focus more on getting the pandemic under control, many oncology healthcare workers across West Africa were redeployed to the COVID-19 frontline [11]. Social distancing measures, movement restrictions, including curfews and lockdowns, and the heightened fear of the transmission and spread of the COVID-19 infection, all these measures further hindered healthcare providers from providing diagnostic services while also deterring patients from accessing available services [11,27]. There was further the concern that cancer patients were at an increased risk of severe COVID-19 disease, hospitalization, and death, hence the suspension or downscaling of most relatively low-to-moderate cancer care services, including prostate cancer early diagnostic practices in this region. For example, delays in scheduling diagnostic and imaging procedures were reported in Ghana [28]. In Cote D’ Ivoire, recent evidence also revealed a 32% reduction in prostate cancer-related diagnostic services [29]. In addition to these restrictive measures, factors such as inadequate infectious disease control plans; limited resources, including protective gears and COVID-19 vaccines; and the emergence of new variants of the virus further complicate any meaningful effort to restore prostate cancer diagnostic services at least to the pre-pandemic level in the region. The continued postponement of early diagnostic services will not only lead to late disease presentation but also an alarming increase in emergency diagnosis which could overburden the health system. As the West African population is already at an increased risk of late-stage prostate cancer, further delays in prostate cancer diagnosis will come with severe prognostic implications.

In this article, we make a case for the operationalization of the World Health Organization (WHO) guide to early cancer diagnosis to strengthen the capacity for prostate cancer early diagnosis in the West African region. First, we present a snapshot of the guide and then illustrate a realist research approach to facilitate the evaluation and analysis of existing service capacity for early prostate cancer diagnosis in the region. Using realist theory, we ultimately illustrated how participatory research and evidence-based co-creation can be leveraged to develop COVID-19 responsive and context-specific strategies to improve timely prostate cancer diagnosis in the region.

## World health organization 2017 guide to cancer early diagnosis

In 2017, the WHO established a comprehensive guide to serve as an operational framework for early cancer diagnosis [30]. The ultimate goal was to support countries to build capacities for early cancer diagnosis and strengthen overall national comprehensive cancer management plans as a critical step for achieving target 3.4 of the 2030 United Nations sustainable development goals. The guide identified elements that drive early cancer diagnosis to include community empowerment and engagement, improving health literacy, access to primary care, increasing diagnostic capacity, strong referral pathways, patient-centered management, and access to timely treatment. Three key steps are articulated for strengthening capacity for early cancer diagnosis: 1) increasing awareness and access to care, 2) clinical evaluation, diagnosis, and staging, and 3) access to cancer treatment. While there is no one-size-fits-all approach to implementing these recommendations, successful operationalization and implementation of the guide can both reduce delays in cancer diagnosis and advanced-stage presentation.

### Step 1— increasing awareness and access to care (or patient interval)

Symptom appraisal, that is patient’s ability to spot a bodily change and discern the need to discuss changes with a healthcare provider, and health-seeking behavior, referring to the “action” of visiting a healthcare provider to discuss and assess bodily changes or symptoms, are the essential elements that define “awareness and accessing care” [30]. To make these decisions, patients must be in a position to “recognize prostate cancer symptoms, understand the urgency of these symptoms, overcome fear or stigma associated with prostate cancer and be able to access primary care” [30]. Patients’ inability to make early discernments and the lack of accessible, affordable and culturally responsive primary care service can result in delays with more men presenting to emergency care with advanced or terminal stage disease.

In our recent study, we showed that lack of prostate cancer knowledge and symptom awareness are the major determinants of poor health-seeking behavior (at the individual level) [25]. Men with a low prostate cancer awareness were most likely to prolong symptom appraisal interval, interpret symptoms wrongly, and delay help-seeking [31]. Governments and mass media have huge roles in promoting prostate cancer literacy in the region by fostering community-based health promotion initiatives, including educational campaigns and regular media enlightenment programs. Traditional leaders and faith-based institutions are also critical for championing prostate cancer awareness, particularly in local communities, given the strong ties between religious groups and traditional leaders and their communities. This offers potential benefits of bringing health education to people within their belief contexts [32].

Limited access to care can also result from fatalistic beliefs and dysfunctional social/cultural norms. In Nigeria, fatalism has led many men to seek alternative care either from “faith-based centers” or traditional healers [25]. Fear of embarrassment, stigmatization, financial constraints, poor transport systems, past unpleasant hospital/medical experience, and lack of family support are other factors that have impacted negatively on men’s health-seeking behavior and restricted access to care [25]. Efforts to increase early prostate cancer diagnosis in West Africa must include culturally relevant strategies for mitigating these barriers.

### Step 2— clinical evaluation, diagnosis, and staging (or diagnostic interval)

Diagnostic interval is defined by three key components: accurate clinical diagnosis, diagnostic testing and staging, and referral for treatment [30]. Diagnostic interval begins with a suspecting patient entering the health system for evaluation. Patients with suspicious results are subjected to appropriate laboratory or imaging investigations and pathological confirmation to determine the presence or absence of cancer. Once a diagnosis is confirmed, patients undergo further examination to ascertain the disease stage and ultimately the best treatment option.

Delays can occur at different levels along the diagnostic pathway. Delays or “access delays” may arise due to human factors such as a patient’s refusal or delay to utilize recommended referrals from their healthcare providers for investigations and further consultations with a specialist where there are perceived symptoms suggestive of prostate cancer [33]. Financial or geographical barriers can deter patients from pursuing recommended referrals. For example, the high cost of living in West Africa and increasing pressure to cater to one’s family have resulted in low prioritization of health and delays in seeking care [25]. Good communication and adequate follow-up can mitigate the chances of a delay or non-pursuance arising from a patient. Doctors can create time to discuss not only the steps involved with the patients but also the need and urgency of pursuing the recommended tests. Having follow-up mechanisms in place, for example, by involving community health workers and patient navigators or by using mobile devices, including text messages, can also spur patients to act even in the face of challenging situations [30].

Diagnostic delays can also result from not initiating appropriate investigations by the healthcare provider [33]. “Doctor-related” diagnostic delays are primarily due to time constraints or lack of the requisite clinical evaluation skills and expertise, such as the ability to appropriately perform digital rectal examination or prostate biopsy [30]. One major strategy to mitigate doctor-related diagnostic delays in the West African region is to improve capacity at the primary care level or first contact point. Another major barrier to a timely prostate cancer diagnosis is health system-related, arising primarily due to factors that impede a healthcare provider’s ability to initiate and execute appropriate diagnostic evaluation or issue timely referrals [33]. There is, therefore, a definite need for establishing clinical examination protocols and algorithms, and routine training on patient evaluation. Greater efforts are needed to ensure adequate investment in early detection modalities, including priority technologies, and increasing capacity for pathological and imaging services in the West African region. In addition, by increasing the oncology health workforce and establishing clear referral pathways, health authorities can empower providers to execute timely and effective prostate cancer diagnoses [30].

The WHO list of priority medical devices for cancer management recommends prioritizing “basic high-impact, low-cost technologies” [30]. This suggests that secondary care levels across West Africa should be prepared to undertake services beyond basic diagnostic tests, such as prostate biopsy and diagnostic staging. Currently, about 1 in 5 men are diagnosed by clinical examination alone; at the same time, up to 55% of patients lack complete TNM stage documentation [10]. The implication is that only a handful of patients receive full diagnostic workup and staging, a proportion far less of current international recommendations.

Significantly, diagnostic delays can be bridged with strong referral mechanisms that coordinate critical health services, such as general practice, urology, pathology, radiology, psycho-oncology, etc., along the diagnostic pathway, across different levels of primary, secondary, and tertiary healthcare in the region [30]. According to WHO, the ultimate goal is to “minimize delays in care and provide integrated, people-centered care through (1) coordinated, efficient referral systems that facilitate access, improve communication and reduce unnecessary visits; (2) linking primary care and outpatient specialty care to advanced diagnostic and treatment services; and (3) effective communication between patients, families, and providers, encouraging patient participation and shared decision making” [30]. Efforts should be made towards establishing diagnostic algorithms, quality control measures, referral and counter-referral guidelines, information transfer channels, and medical record systems to facilitate the delivery of time-sensitive, accurate, and high-quality diagnoses [30].

### step 3— accessing treatments

Early prostate cancer diagnosis should ultimately culminate in men with prostate cancer being able to access high-quality, culturally responsive, and affordable treatment services at best within one month of confirming prostate cancer diagnosis [30]. Depending on the disease stage and other host factors, including age, comorbidities, personal preferences, and cost, men with prostate cancer might be placed on active surveillance/watchful waiting (with no treatment) or receive a single/multimodal course, with definitive intents, such as radical prostatectomy and radiation therapy (with external beam or brachytherapy), or non-curative therapies, such as hormonal (androgen deprivation) therapies, radiation, and chemotherapy [34,35].

Factors that could impact the timely initiation of prostate cancer treatment, consequently resulting in delays or abandonment, include high treatment cost and out-of-pocket spending, which also are the leading causes of the financial burden associated with healthcare in the West African region. The high cost of treatment stems largely from a fewer number of surgeons and physician oncologists (~2 surgeons per 100,000 men) to scarcity of therapeutic modalities, including radiotherapy machines and androgen-suppressing and chemotherapeutic drugs [13]. Unfortunately, these products are only available upon importation from other countries. Out-of-pocket spending, on the other hand, is primarily due to the lack of health coverage, such as national health insurance, or more radical measures including government reforms to guarantee access to affordable healthcare coverage for everyone. In Nigeria, for example, health economists have estimated the average per-person lifetime prostate cancer-related cost to be in the realm of USD 27 500. With the growing economic crisis in West Africa, two-thirds of men with prostate cancer will delay treatment due to their inability to pay for care [24,36,37].

Prostate cancer treatment services are not only nonexistent in many public hospitals and healthcare facilities, but also, where available, are largely substandard and centralized [10,23,36,37]. For instance, the first brachytherapy treatment in Nigeria using low dose radioactive iodine 125 permanent seed implant was only reposted in 2020 [38]. In Ghana, where there is evidence of functional radiotherapy center, the proportion of men with nonmetastatic disease receiving curative radiotherapy can be as high as 56% [23]. The travel costs and time required to seek care, particularly for men in rural and remote settings, will add to the disease burden, thus precluding men from accessing treatment promptly [39]. Treatment delays may further arise due to incessant strike actions, infrastructural decay, previous medical experiences, fears and uncertainties associated with prostate cancer treatment, embarrassment, stigmatization, and poor communication between patients and their doctors [25,39].

The WHO further recommends prioritizing and decentralizing basic high-impact prostate cancer treatment services while mitigating out-of-pocket spending using means such as national health insurance [30]. Prostate cancer diagnosis and the associated treatment are stressful life events with strong implications for men and their relatives. Routine counseling should be made available to assist men in coping with the psychosocial demands of prostate cancer diagnosis and prepare them for the journey ahead [40,41]. Community health workers, social support groups, civil societies, patient advocates, and cancer survivors can promote early prostate cancer treatment by using their voices to dispel age-long myths, misconceptions, and false narratives surrounding prostate cancer in their communities. Again, treatment delays and loss to follow-up can be reduced with effective communication between patients and their service providers and by integrating community health workers and patient navigators in the cancer care pathway [30].

We conclude this section with the following excerpt from WHO guide to cancer early diagnosis:

“Improving early diagnosis requires health system investment at all facility levels and across WHO health system building blocks – health workforce, access to priority technologies, health financing, health information systems, leadership and governance, and service delivery – according to local capacity….” (WHO; p.22).

## A realist research approach to developing practical and context-appropriate models for strengthening capacity for early prostate cancer diagnosis in West Africa

As health systems grapple with effective and evidence-based methods for the re-escalation of early diagnostic practices globally, the realist research approach can facilitate the development of COVID-19 responsive and context-specific models to strengthen capacity for early prostate cancer diagnosis in West Africa. The realist methodology is increasingly gaining momentum as a more pluralistic approach to exploring hidden contextual forces and dynamic milieu surrounding complex public health and healthcare challenges [42]. It seeks to support researchers and stakeholders to generate “explanatory models” and “program theories” that unpack the plausible mechanisms undergirding effective interventions [42]. Realist evaluation is a theory-driven approach that offers an in-depth approach to understanding *how, for whom, in what circumstance, and to what extent* programs and services work [42,43]. The context–mechanism–outcome (CMO) configuration defines the structural units of the realist methodology [44]. The argument is that in any given social structure, health services work, for example, when key actors and stakeholders appreciate what and how relevant service components ***interact*** (M) with ***evolving time, conditions, and circumstances*** (C) to create the desired ***changes*** (O) [42].

**Figure 1:**
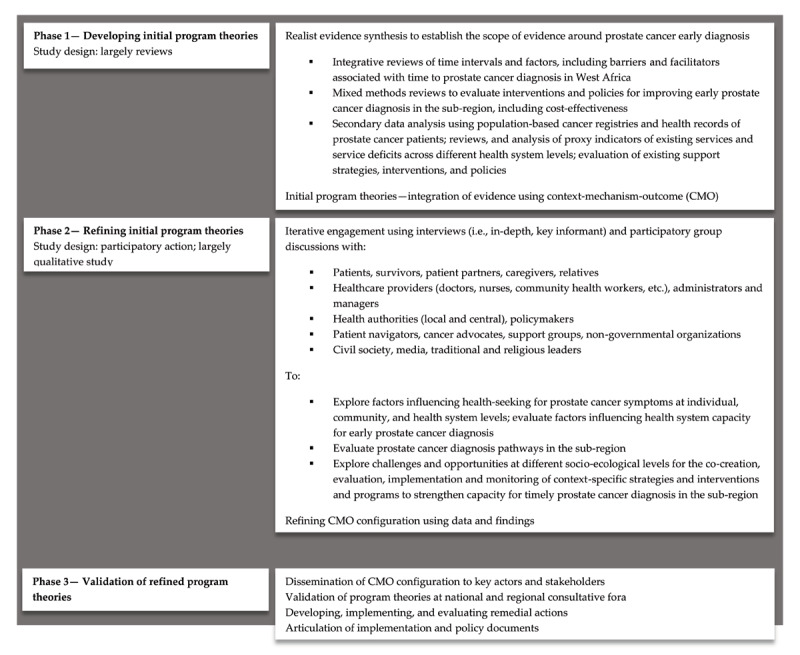
Illustrating a realist research approach for developing a practical and context-specific model to strengthen capacity for early prostate cancer diagnosis in West Africa.

Realist methodology embraces the mixed-methods paradigm, engaging more iteratively and purposefully not only with data and the literature but also with key actors and relevant stakeholders to articulate, test, and refine program theories that account for how and why programs work while identifying, implementing, and evaluating effective solutions [45]. In contrast with the traditional health research paradigm, the realist methodology is inherently pluralistic and flexible, embracing both qualitative and quantitative data, across formative and summative, prospective and retrospective perspectives, in the quest to understand and mitigate complex health issues [46]. In the context of early prostate cancer diagnosis in West Africa, this would entail first establishing the theoretical landscape of patient navigation/journey followed by a situation analysis of existing services, including barriers and delays in diagnosis (Figure 1). This information will drive the formulation of initial program theories that will isolate the contextual factors supporting or hindering early prostate cancer diagnosis in the region and identify where and how to target possible interventions. Such information will also be used to develop, implement and evaluate strategies and interventions that will be responsive to the changing times.

### Developing initial program theories

The major entry point for understanding the diversity and complexity of factors surrounding early prostate cancer diagnosis in West Africa while exploring opportunities and mechanisms for remedial actions is to establish the landscape of the existing literature. This is the first step in the formulation of initial program theories. A critical component of research at this stage involves realist and meta-narrative syntheses of the literature on the multi-dimensional impacts of the COVID-19 pandemic on patient navigation/journey. This requires multiple yet complementary reviews using a contingent design to answer critical questions [47]. As results emerge from the initial exploratory review of the literature, subsequent reviews could be commissioned to answer emerging questions. Such reviews will explore diagnostic time intervals, including barriers and facilitators to early prostate cancer diagnosis in the region, whether they be individual, sociocultural, structural, or system-wide factors. A multimethod analysis of peer-reviewed literature is also required to evaluate existing strategies, including interventions, programs, and policies for improving early prostate cancer diagnosis in the sub-region. At this stage, a review of secondary data, including health facility data and records of prostate cancer patients, to summarize [proxy indicators of] existing services and service deficits across different health system levels is essential for a grounded insight on current service capacity across West Africa. Findings at this stage will inform the initial program theories that will explain, using the C-M-O configuration, the dynamic circumstances, and conditions surrounding prostate cancer diagnosis in the region while exploring potential causal pathways.

### Refining initial program theories

A multi-level situation analysis of existing service capacity for early prostate cancer diagnosis in the region is critical to developing and implementing context-specific and evidence-based solutions. The goal at this stage is to evaluate the existing capacity for early prostate cancer diagnosis and understand individual, interpersonal, community, and health system-level factors, including barriers, impacting patients’ journeys along the diagnostic pathway. In West Africa, evidence has shown that men often enter the diagnostic pathway from different points, mainly through emergency admission [25]. Therefore, studies at this stage should include qualitative exploration of the individual- and community-level factors influencing health-seeking behaviors and access to care in men with prostate cancer from different perspectives. At the healthcare level, efforts should be directed at identifying the structural and organizational factors that impact health system capacity for timely prostate cancer diagnosis. Strengthening capacity for early prostate cancer diagnosis in West Africa requires (1) investing massively in oncology workforce and routine training; (2) investing in pathology and imaging; (3) establishing guideline-concordant care with well-defined service protocols, referral pathways, and quality control mechanisms; (4) integrating medical records system; and (5) promoting integrated and patient-centered care [10,12,13,30]. In this regard, emphasis should be placed on examining facilities’ capacities, needs, and preparedness across primary, secondary, and tertiary levels. At this stage, analyzing data by sociodemographic characteristics is vital to identify and address inequalities surrounding access to early diagnosis.

Research at this stage should be participatory. Studies involving different actors and stakeholders including patients, survivors, patient partners, caregivers, relatives, patient navigators, cancer advocates, support groups, non-governmental organizations, healthcare providers (doctors, nurses, community health workers, etc.), health administrators and managers, health authorities (local and central), policymakers, civil society, mass media, traditional institutions, and religious leaders are likely to yield useful results [48]. Key stakeholders should be invited to evaluate challenges and opportunities for co-creating, implementing, and evaluating relevant solutions to strengthen capacity for timely prostate cancer diagnosis in the region. Data emerging at this stage are critical for refining and validating the program theories.

### Validating the program theories

The ultimate purpose of a realist-driven evaluation is to generate and validate context-specific models (or “program theories”) structured around the context-mechanism-outcome configuration to isolate dynamic and emerging factors that trigger the right mechanisms to create desired outcomes. The anticipated outcome in this context is improved capacity for early prostate cancer diagnosis in West Africa. The program theories that have emerged as products of multiple reviews, evaluations, and analyses of current service capacity in the region will inform quality improvement interventions. Collaborative and iterative efforts are required among key actors and stakeholders to validate the program theories and develop quality improvement interventions that can improve early prostate cancer diagnosis in the region [49]. The Prostate Cancer Transatlantic Consortium (CaPTC) and the African Organization for Research and Training in Cancer (AORTIC) can offer excellent platforms for validating the program theories. As leading bodies of researchers, experts, and stakeholders addressing the burden of [prostate] cancer in the African continent, these organizations are suitably positioned to validate the program theories and generate ideas including strategies and actions to be implemented to strengthen early prostate cancer diagnosis in West Africa based on the validated program theories [49,44]. Critical at this point is articulating action plans and policy documents to guide health systems and service providers in implementing recommended change strategies while supporting governments and health authorities in making relevant policies. This can be achieved by setting up a technical working group that will foster collaborative engagements with researchers, clinicians, patients, health authorities, policymakers, and other key stakeholders in line with the agreed actions and targets.

## Conclusions

As the burden of prostate cancer continues to increase in many resource-constrained societies, timely diagnosis remains a major weapon against late-stage presentation and deaths from the disease. Concerted efforts are needed to ensure that existing services supporting timely prostate cancer diagnosis do not collapse in the aftermath of the COVID-19 pandemic. In this article, we have shown how a realist research approach can be leveraged to develop practical and context-responsive models or “program theories” to strengthen capacity for early prostate cancer diagnosis in West Africa along the essential steps of the WHO guide to early cancer diagnosis. We anticipate that this article will encourage organized and collaborative efforts that will lead to developing and implementing timely solutions to strengthen the capacity for early prostate cancer diagnosis in West Africa.
